# For You and Your Baby (4YYB): Adapting the Centers for Disease Control and Prevention’s Text4Baby Program for Saudi Arabia

**DOI:** 10.2196/resprot.5818

**Published:** 2017-02-28

**Authors:** Soha Bahanshal, Steven Coughlin, Benyuan Liu

**Affiliations:** ^1^ Department of Computer Science University of Massachusetts Lowell Lowell, MA United States; ^2^ Department of Clinical and Digital Health Sciences College of Allied Health Sciences Augusta University Augusta, GA United States

**Keywords:** Saudi Arabia, telemedicine, text messaging, health knowledge, attitudes, practice, pregnant women

## Abstract

**Background:**

Poor birth outcomes in the Kingdom of Saudi Arabia (KSA) have been found to be partially due to missed prenatal appointments as well as lack of knowledge of healthy pregnancy behaviors.

**Objective:**

The objectives are to summarize birth outcomes and the antenatal care system in KSA, summarize research related to the US Text4Baby mobile health program, and outline the development of an Arabic version of the Text4baby app, For You and Your Baby (4YYB).

**Methods:**

First, birth outcomes, health care access, and smartphone usage among Saudi Arabian women are reviewed. Next, the current evidence behind Text4Baby is described. Finally, a plan to develop and test 4YYB is proposed. In the plan, studies will need to be conducted to determine the effectiveness of 4YYB in educating pregnant Saudi women on healthy knowledge and behaviors. This will create an evidence base behind 4YYB before it is launched as a full-scale public health effort in KSA.

**Results:**

The KSA offers public medical services but remaining challenges include poor birth outcomes and health care access barriers. An estimated 73% to 84% of Saudi women of child-bearing age use smartphone social media apps. A total of 13 published articles on Text4Baby were identified and reviewed. Due to design limitations, the studies provide only limited evidence about the effectiveness of the program in increasing healthy pregnancy knowledge and behaviors. To be useful for Saudi women, the educational messages in 4YYB will need to be translated from English to Arabic and tailored for cultural norms.

**Conclusions:**

Developing the 4YYB Arabic-language app for use by pregnant Saudi Arabian women based on Text4Baby is a viable approach, but a rigorous study design is needed to determine its effectiveness in improving healthy pregnancy knowledge and behaviors.

## Introduction

### Background

Although the health care system in the Kingdom of Saudi Arabia (KSA) is undergoing a dramatic expansion and there is a government-sponsored health system, not all health outcomes are being impacted optimally. There are remaining challenges with poor birth outcomes [1], and Saudi women still experience health care access barriers. Since a majority of Saudi women are users of smartphone social media apps [2], a mobile health platform for delivering messages to increase healthy pregnancy knowledge and behaviors could help address this important public health issue.

Text4Baby is a mobile health text messaging (short message service, SMS) program developed in the United States that has been disseminated nationally [3]. While Text4Baby has received much praise [3], the program has been criticized for lacking a scientific evidence base [4]. The aim of this paper is to discuss how we plan to adapt the Text4Baby program to serve Saudi Arabian women in an Arabic-language mobile app, For You and Your Baby (4YYB). The objectives of this paper are to summarize birth outcomes and the antenatal care system in KSA, summarize research related to the US Text4Baby mobile health program, and outline the development of an Arabic version of Text4Baby, 4YYB. Our hypothesis is that there are poor birth outcomes and obstetrical care in KSA, and a 4YYB program could improve this situation.

### Poor Birth Outcomes in Saudi Arabia

KSA is currently challenged with unacceptably high rates of poor birth outcomes and neonatal complications in women. This includes issues with infants born with very low birthweight (VLBW), spontaneous preterm birth (SPTB), obstetric complications, unacceptable neonatal mortality rates, and other medical issues. This section will review the evidence regarding risk factors for poor birth outcomes in KSA.

Infants born at VLBW (defined as less than 1500 g) are at high risk for infant mortality, morbidity, and neurological developmental disabilities [5]. A 1994 case-control study of SPTB in Saudi Arabian women found associations with several risk factors including first or second trimester vaginal bleeding, previous preterm birth, consanguinity, low maternal body mass index, shorter interpregnancy interval, and inadequate prenatal care [6]. The authors concluded that “Awareness of such risk factors is essential in planning public education programs and in considering appropriate perinatal care options for women at potentially higher risk for preterm delivery” [6]. Another 1994 study of 880 pregnant Saudi Arabian women attending appointments at 75 primary health care centers found that 15.8% had experienced previous obstetric complications during their pregnancies and 12% experienced complications during delivery [7].

A 2001 study at King Abdulaziz University Hospital (KAUH) estimated the incidence of VLBW at 0.52% (133 out of 25,753 live births) over a 10-year period [1]. VLBW infants experienced an early neonatal mortality rate of 22.8% (21 out of 92 infants of gestational age 22 to 26 weeks) with 1 additional late neonatal death (gestational age 27 to 31 weeks), resulting in a total neonatal mortality rate of 23.9% (22 out of 92). [1]. In this study, the authors looked at maternal characteristics associated with 2 groups of VLBW infants with a gestational age of less than 32 weeks and found that the number of antenatal visits was only 2 on average [1].

A 2010 study of the prevalence of SPTB in Jazan, KSA, estimated it at 8.24% (34 of 420) [8]. The authors noted that this was “high compared to that in other cities in KSA and other developing countries” [8]. These authors identified 22 significant risk factors for SPTB in multiple regression analysis [8]. They recommended early identification of high-risk women for further management, which could bring about a decrease in both neonatal complications and health care expenditure [8]. However, it is important to recognize that Saudi women who do not encounter the health care system are unlikely to be identified as high-risk and receive necessary services.

Other authors have identified overlapping sets of risk factors for poor birth outcomes in KSA [5]. A 2014 study of anemia in 31 women of reproductive age attending an obstetrics and gynecology outpatient center at a university in Saudi Arabia reported that 64.7% of anemic respondents were pregnant, so anemia may be one of the risk factors for poor birth outcomes in KSA [9]. Another study in 2014 of 1182 postpartum women at a university hospital in Riyadh, Saudi Arabia, found that while 80% were aware of the negative effects of environmental smoke on the fetus, their knowledge of the specific effects on the fetus was lacking [10].

Overall, many of these articles point to the lack of prenatal care as a key risk factor [6]. As seen with the 2014 studies, health care interventions focused on eliminating anemia in pregnant women and counseling them on smoking cessation could be completed in the context of prenatal care. However, these are missed opportunities if pregnant women do not present for prenatal care. For example, Almalki studied reasons for missed appointments at maternal health care clinics in primary health care centers in Riyadh, noting that international rates of missed appointments range from 2% to 30% and are higher in maternal health care [11]. Almalki found that the 2 main reasons that women missed their appointments were lack of adequate tools at the medical clinic to do the examination (such as unavailability of a ultrasound machine, 143/200, 71.50%) and the unavailability of transportation (142/200, 71%) [11]. These barriers to completing a successful prenatal appointment may be enough to dissuade a woman from persevering through them.

### Missed Appointments as a Risk Factor

As described earlier, 1 study quoted an average of 2 appointments in high-risk women prior to a VLBW infant being born, and 1 of the main reasons was lack of the availability of adequate tools for the examination [11]. This impacts the patient on the day the failed appointment takes place but can have future effects. Women who attend an appointment but cannot be served would likely be deterred from overcoming any barriers to attending future appointments, which would increase the risk of poor neonatal outcomes. While prenatal care is universally available, arranging transportation for women in KSA can be difficult and presents a barrier to attending appointments [11].

**Figure 1:**
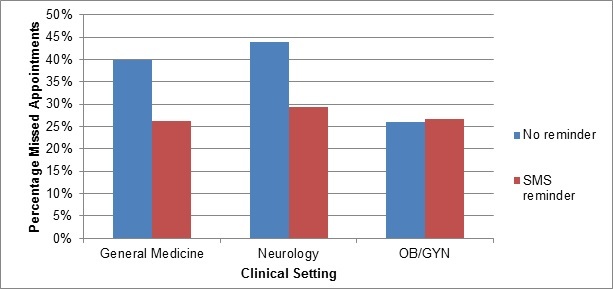
Results of study of clinical appointment nonattendance and short message service use (adapted from Youssef et al [13]).

However, there may be also a cultural component [12]. Missed appointments are a problem in KSA in general, not just in obstetrics and gynecology (OB/GYN) clinics. Noting the high usage of text messaging in Saudi Arabia, Youssef et al studied the effect of sending SMS messages as appointment reminders in KSA compared to no appointment reminders to improve nonattendance [13]. They aimed to improve appointment attendance in 3 clinical settings: general medicine, neurology, and OB/GYN [13]. Figure 1 shows their results.

As shown in Figure 1, rates of nonattendance in the no reminder groups were higher in both general medicine (100/251, 39.8%) and neurology (87/198, 43.9%) compared to OB/GYN (104/350, 29.7%) [13]. The use of the appointment reminder did not improve appointment attendance among the OB/GYN patients, but that may have been because of the other barriers identified in earlier studies, such as lack of transportation [11].

In summary, use of prenatal services by pregnant women in KSA appears to be low, and this contributes to poor birth outcomes. Using text messaging could improve appointment compliance, but given the barriers listed to completing appointments, a technological application that actually provides knowledge and guidance to pregnant women might be more helpful than 1 that simply provides appointment reminders.

### Antenatal Care in Saudi Arabia

The Saudi Arabian health care system is divided into a government sector, which offers free care, and a private sector, where fees are paid for care [14]. The private sector provides all levels of care, and the public sector is further divided into Ministry of Health–run facilities, which provide all levels of care, and facilities run by other agencies, which also provide all levels of care [14]. These other agencies include teaching hospitals, military health facilities, and school health units, among others [14]. More recently, Saudi Arabia has been transitioning from a curative medicine model to more public health and prevention, and this is seen by its enhancement and expansion of primary health care [14]. Although the Saudi Arabian health care infrastructure continues to expand and mature, it currently still faces challenges with “health workforce, financing and expenditure, changing patterns of diseases, accessibility to health care services, introducing the cooperative health insurance scheme, privatization of public hospitals, utilization of electronic health (e-health) strategies and the development of a national system for health information” [14].

Because of the availability of free care, there are few economic barriers to health access for pregnant women [14]. Public hospitals and medical complexes such as the Ministry of National Guard Health Affairs (NGHA) in Jeddah allow for women to receive health care at no cost [15]. This hospital serves families of those in the National Guard and has OB/GYN clinics to serve pregnant women.

The NGHA clinic is similar to other public clinics in that when women are found to be pregnant, they are enrolled in a pregnancy health program. In this program, the woman is scheduled for a second appointment during the first trimester (at 8 to 10 weeks), an appointment during the second trimester (at 18 to 20 weeks), and appointments in the third trimester at 28, 32, 35, 37, 39, and 41 weeks.

Women are also offered antenatal education classes taught by midwives. Women may decline to participate in these classes. The classes provide videos and pamphlets aimed at increasing pregnancy knowledge. When women are preparing for pregnancy, they are encouraged to take folic acid starting 3 months prior to pregnancy. They are told to continue folic acid until 16 weeks and then switch to iron tablets, calcium, and a multivitamin. Even though clinics like the one at NGHA are very accessible, pregnant women in KSA have reported barriers to accessing health care [11]. As described before, a 2012 study reviewed reasons 250 maternal health patients missed appointments and found that the most frequent reasons were lack of supplies and medical equipment such as ultrasound machines, the unavailability of transportation, and lack of respect from the primary health care center staff [11].

### Saudi Women and Mobile Health

**Figure 2:**
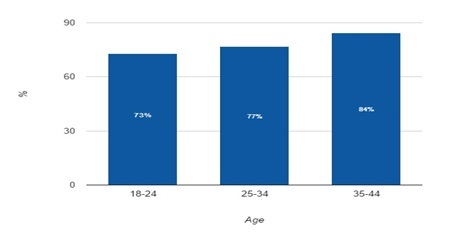
Distribution of Saudi Arabian women’s 2013 smartphone use of social networking apps stratified by age (from Our Mobile Planet).

A large proportion of Saudi Arabian women of child-bearing age use smartphones and are adept at texting. The Our Mobile Planet online dataset provided by Google shows that 73% of Saudi Arabians regularly use smartphones [2]. Figure 2 shows the 2013 distribution of Saudi Arabian women’s smartphone use specifically of social networking apps (as Text4Baby was characterized) stratified by age. Per the figure, the use in this group of social networking apps is between 73% and 84%.

Because of the high adoption of mobile technology in KSA, a messaging application that goes beyond the walls of the clinic could deliver important information these women are missing by missing their appointments. Nutrition information, guidelines about attending prenatal care visits, and recommendations for adopting certain health behaviors (such as getting a flu shot or quitting smoking) could be provided to these women without the need of a clinic appointment. A similar rationale was behind the development of the Text4Baby program in the United States, which was aimed at underserved pregnant women who may not have had access to health care.

### The Text4Baby Program

Text4Baby is a texting health education system developed in an effort led by the US Centers for Disease Control and Prevention (CDC) to reach low socioeconomic status (SES) pregnant women who are thought to be at high-risk for negative birth outcomes such as VLBW and SPTB [16]. This system was initially developed and piloted in 2011 and 2012 [17-19]. The main themes included in the messages are information about healthy weight, nutrition, and exercise; information about depression and anxiety; encouragement and information for seeking care; breastfeeding information; roles of partners in pregnancy; the celebration of pregnancy; prevention strategies including vaccinations, contraception, and sudden infant death syndrome prevention; and information on avoidance of toxic exposures [20].

Currently, to participate in Text4Baby, pregnant women sign up on the Text4Baby website [21]. Participants enter personal information, such as when the baby is due and their cell phone numbers [21]. Each week, the pregnant woman receives pregnancy health messages appropriate for her week of pregnancy. Text4Baby offers colorful and attractive apps for both iPhone and Android [21].

The Text4Baby site offers true stories from users who endorse the support and health education that Text4Baby has provided [21]. The CDC and state health departments are actively promoting Text4Baby [22]. Text4Baby has been adapted for a Russian population [23] and has been tested extensively in the US military [16]. Therefore, it appears to be an excellent system to adapt for the KSA pregnant population.

However, Text4Baby has also been criticized for redundant messages [20] and for lacking an evidence base [4]. The next section will review the current evidence regarding the public health implications of the Text4Baby program.

## Methods

Although the program was launched in 2010, evidence regarding the effect of the messages on the knowledge and behavior of pregnant women remains largely elusive. This study will use a traditional or narrative type of literature review, which critiques and summarizes a body of literature and draws conclusions about the topic [24,25]. This type of literature review is important for identifying both gaps and inconsistencies in a body of literature on a scientific topic but requires an extremely focused topic [24,25]. This can be contrasted to the systematic review and meta-analysis approach, which relies on combining findings from many studies of similar study design conducted on the same topic [24,25]. Because Text4Baby was launched relatively recently and because the study designs and research questions in published reports are diverse, these approaches could not be taken at this time with this topic.

Articles were reviewed if they met the following criteria: (1) the main topic of the article was the Text4Baby program and (2) the article was in English. Articles were originally identified by searching for Text4Baby in Google Scholar. An initial search generally returns approximately 670 results; this was conducted first in June 2014 and then periodically over the next 2 years, with the most recent search being conducted on August 5, 2016. Each entry was reviewed for meeting qualifying criteria, and each article that was found to meet qualifying criteria underwent a source review to identify cited articles that met qualifying criteria.

## Results

### Overview

The literature review resulted in the identification of 14 articles directly related to Text4Baby. Only 7of these articles [16,18,20,26-30] represented actual studies; the others described the program [3,4,17,31] or described a study that was going to take place [19,23]. Of the 7 that described actual studies, 2 focused on the American military population [16,28], 1 was a pilot study [18], 2 focused on the health literacy of Text4Baby participants [26,27], and 2 focused on the content of the messages [20,29].

### Pilot Study

The earliest study published was a pilot study conducted with pregnant women in Fairfax County, Virginia, who presented for care at their Virginia health department. Participants were randomized to receive Text4Baby plus usual care or usual care [18]. Participants were surveyed at baseline and at approximately 28 weeks of the baby’s gestational age [18]. There was a 73% retention rate, and results were determined using multivariate analysis [18].

Unfortunately, the questions asked in the survey were not related directly to Text4Baby messages and instead were copied from surveillance questions: “The variables for behavioral outcomes were derived from existing, validated instruments, including the Behavioral Risk Factor Surveillance Survey and National Health and Nutritional Examination Survey” [18]. Therefore, the questions were not worded to directly measure if any specific messages in Text4Baby were successful at imparting knowledge or changing behaviors. For example, one of the questions was “During the last 3 months, about how many servings of fruit did you have in a day?” However, there was no corresponding message or set of messages in Text4Baby that targeted changing the behavior of how much fruit the pregnant woman should eat.

Because of this mismatch, the results of the pilot study were hard to interpret. First, the groups were not comparable at baseline. For example, at baseline, the proportion who agreed with the statement “Eating 5 or more fruits and vegetables per day is important to the health of my developing baby” was about 71% in the control group versus 56% in the Text4Baby group, suggesting that either there was a randomization problem or the authors did not use an intent-to-treat analysis and simply removed the Text4Baby dropouts from their analysis [18]. Next, the authors performed bivariate statistical tests between both groups but did not find differences [18]. Further, because of a lack of an attention control [32], it was not possible to have the usual care only group, which was the control group, drop out, so rate of dropping out of using the app could not be compared [18].

### Studies of Health Literacy

The next study published was on the health literacy of users of Text4Baby in the Atlanta area [26]. The reason this was a focus of study is that Text4Baby was aimed at a low health literacy population. Their results suggested that those who successfully self-enrolled in Text4Baby were already more health literate than those who did not, which prompted the authors to recommend the need for additional outreach efforts to enroll low literacy women [26]. The same team later studied factors related to the enrollment process and reception of Text4Baby and found that even though there was a high interest among public health practitioners to use Text4Baby to help underserved populations, there remained challenges to making sure women with significant disadvantages could enroll and receive uninterrupted messages [26]. Currently, it seems that the Text4Baby program is not serving the low literacy populations it was aimed to serve adequately, and public health efforts need to be employed to connect underserved women to the Text4Baby program.

### Studies of Health Messages in Text4Baby

Lewkowitz and colleagues produced first a conference abstract and then a paper comparing the content of Text4Baby messages with messages from other pregnancy apps, such as the one sponsored by the American College of Obstetrics and Gynecology and March of Dimes [20,29]. Their analysis suggested that Text4Baby could improve by decreasing the number of messages about obtaining medical care and increasing the number of messages about healthy eating, normal weight gain, exercise, and nutrition without increasing the overall number of messages [20]. The paper later also recommended that Text4Baby replace messages on recruitment with messages about normal pregnancy symptoms, fetal development, and postpartum contraception [29].

### Studies of Text4Baby in Specific Populations

Two studies focused on the American military [16,19] and 1 on women in a homeless shelter [30]. The American military studies were headed by the same researcher who did the pilot study in the Virginia health department cohort, and therefore, the results are similarly difficult to interpret. The first military study was designed similarly to the pilot study—using surveillance questions in the data collection and not using an attention control for the control group [19]. Although the number of participants was significantly larger than the pilot study, the same issues plagued this study, the main results are presented with 1 *P* value, and it is not clear what is being compared in this analysis [19].

Ultimately, due to these design issues with the study and the analysis, it was not possible to determine from the analysis whether Text4Baby increased healthy pregnancy knowledge or was associated with changes in behaviors. The authors did not provide an interpretation of their results in their abstract and said in their discussion that Text4Baby appears to improve “specific targeted beliefs” such as those about the importance of prenatal health care, risks of alcohol use, and importance of prenatal vitamins [28] but did not reference the findings in their analysis to which they were referring.

This military study was followed by another one with the same research team about the dose-response effect of Text4Baby [16]. Again, this study suffered from the same design issues, but authors were able to report that the more messages women received through Text4Baby, the lower likelihood they had of self-reported alcohol consumption [16]. There were no other particular findings reported speaking to the effect on knowledge and behavior change due to Text4Baby.

The third study of a particular population focused on enrolling pregnant mothers in a homeless shelter into Text4Baby [30]. This study did not focus on knowledge and behavioral change; rather, it tried to implement the findings from the studies on health literacy by actively facilitating enrollment in Text4Baby by a low literacy, underserved population [30]. Researchers found that the facilitative, on-site support provided to these homeless pregnant women to help them enroll and use Text4Baby was successful at increasing enrollment and retention rates [30].

### Analysis of Existing Evidence

Currently, it has been shown that women who enroll in Text4Baby have higher health literacy and enrolling lower literacy women will require facilitative support [26,27,30]. However, studies of the efficacy of Text4Baby in increasing healthy pregnancy knowledge and behaviors has not been clearly demonstrated. Although 1 military study shows that women decreased their alcohol intake [16], this is not a problem in Saudi Arabia, where alcohol intake is illegal. Therefore, it is currently unclear how Text4Baby impacts healthy pregnancy knowledge and behaviors.

This particular aspect of the development of the Text4Baby program has been criticized. Before the launch of Text4Baby, there was a lack of evidence supporting the claim that Text4Baby would be effective in changing knowledge and behavior [4]. Even though Text4Baby has now been widely implemented, this evidence base is still modest. In all, 2 studies reviewed [26,27] found that Text4Baby is not currently reaching the low literacy population it is targeting and 1 study showed that on-site enrollment may improve adoption in this population [30].

However, Text4Baby has not been consistently shown to improve pregnancy health knowledge and increase healthy behaviors in users, mainly due to errors in the design and analysis of the few existing studies [16,18,28]. Per the review of the message content, it is unlikely that Text4Baby messages are ineffective or even harmful [20,29]. A likelier scenario is that Text4Baby messages actually do increase pregnancy health knowledge and healthy behaviors, but due to lack of a rigorous study design and execution, this evidence is not currently available.

## Discussion

### For You and Your Baby: Text4Baby for Saudi Arabia

The aim of this project is to develop a similar program as Text4Baby aimed at Saudi Arabian pregnant women. The rationale is that this app would be able to deliver information on healthy pregnancy knowledge and behaviors even if these people do not go to a health care facility. Because Saudi Arabian women have widely adopted smartphone apps and social media, it is reasonable to expect that they would accept the dissemination of health information in this medium. A main health behavior that would be targeted for change in the Saudi Arabian app is missing health care appointments. Other behaviors that would be targeted for changes are consistent with those targeted by Text4Baby: smoking cessation, taking pregnancy vitamins regularly, improving nutritional intake, and avoiding toxic exposures such as contraindicated medications.

The results of our review demonstrate that although there are several publications on the Text4Baby program, a strong evidence base does not currently exist demonstrating that Text4Baby is effective at its original intention, which is to increase healthy pregnancy knowledge and behaviors in US women, especially those of low health literacy and SES. This should not dissuade researchers from seeking to adapt and improve this program for other populations, provided that sufficient evidence is gathered prior to launching the program. As described with the Russian effort [23], international partnerships are needed to properly adapt and study different Text4Baby programs before they are implemented. Although there is a lack of evidence of efficacy, the implementation has been highly studied and therefore provides an efficient starting point for 4YYB.

**Figure 3:**
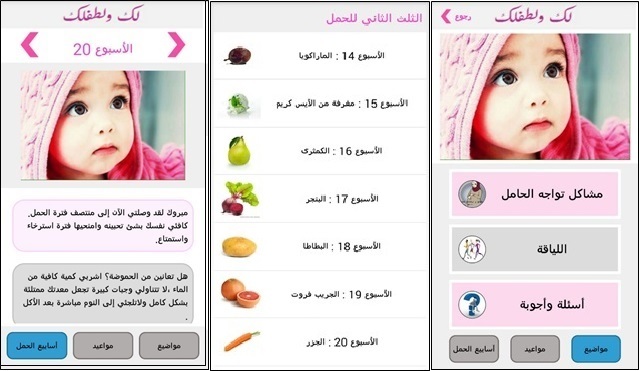
Screen shots from 4 You and Your Baby. Left panel shows message from pregnancy week 20. Center panel shows diagrams of baby’s size at various weeks. Right panel shows dashboard for health information.

We plan to develop a culturally tailored Arabic-language version of Text4Baby and examine its effectiveness in increasing healthy pregnancy knowledge and behaviors in Saudi Arabian pregnant women. The new program, 4YYB, would be a mobile health app that runs on iPhone and Android platforms and provides culturally appropriate health information to pregnant Saudi women. Like with Text4Baby, women could download the app, enter their due dates, and receive health messages appropriate to their week of pregnancy. The app would also include diagrams of the baby’s size at various weeks as well as health information on topics such as exercise during pregnancy (see Figure 3). The goals of the messages in the app would be to encourage women to present for prenatal visits and provide knowledge about healthy pregnancy behaviors that should be provided at prenatal visits, whether or not they attend them. The healthy pregnancy behaviors targeted by the app would be similar to those in the original Text4Baby: healthy nutrition, exercise, prenatal care, celebrating pregnancy, and avoidance of toxic exposures, as described earlier.

Messages would need to be not only translated into Arabic but adapted for the Saudi population. Alcohol is forbidden in Saudi Arabia while on the other hand, hookah smoking is popular. Messages will need to reflect cultural norms and practices and be culturally appropriate.

### Anticipated Use of For You and Your Baby

As described earlier, missed appointments are a problem throughout the Saudi Arabian health care system. This is possibly compounded by the common cultural belief among pregnant women in KSA that they are not sick, so they do not need to attend health care appointments. Further, many pregnant women rely on their families rather than medical professionals when they have symptoms [12]. On the other hand, women in KSA are on average highly educated and tend to actively seek knowledge, especially health knowledge [33]. Also, women of child-bearing age are typically active users of smartphones and social media [2]. As a result, this population will be likely to adopt 4YYB because it delivers health information tailored to their specific week of pregnancy via a smartphone app. Further, a review of Arabic-language apps in Apple’s App Store reveals a lack of apps aimed at this population providing this type of information. We expect the 4YYB app will fill the gap for an Arabic-language app devoted to pregnancy.

### Development of For You and Your Baby

Text4Baby was designed to provide healthy pregnancy information to low-income US residents who may experience access to care barriers [26,27]. Many aspects of the design were evidence-based, such as having an expert panel develop the messages and modifying the app based on results from preliminary focus groups and interviews [3,31]. However, even with these promising elements, early studies of the efficacy of a prototype Text4Baby program in terms of successfully increasing knowledge and healthy behaviors in pregnant women from use of the app were not conducted [4]. Therefore, the actual impact of Text4Baby on these parameters remains unknown.

To avoid this problem in the development of 4YYB, research will first be conducted to determine the efficacy of a prototype 4YYB app in Saudi Arabian populations who may experience health care access barriers and inadequate information about maternal and newborn health. Studies should be conducted of the impact of the messages on users of the public health infrastructure, like those at the OB/GYN clinic at NGHA. As was pointed out by Gazmararian and colleagues, the program is designed to target those with low health literacy [27], and this population would be more likely to use public services.

### Proposed Research

Ultimately, an evidence base for the effectiveness of 4YYB should be developed before it is launched in Saudi Arabia or other Arabic-speaking countries. So far, a randomized controlled trial (RCT) has not been published that demonstrates the efficacy of increasing healthy pregnancy knowledge and behavior in users of the Text4Baby program. In order to do that type of study, a control app providing nonhealth pregnancy messages should be developed. Participants in the study will then be randomized to either the control app group or the 4YYB app group and later followed for increases in healthy pregnancy knowledge and behavior. The use of a control app will offer an attention control [32] and a comparable comparison as to the uptake in knowledge and change in behaviors that take place during use of the app that are attributable specifically to the 4YYB messages.

During the RCT, questions used in the research data collection will be structured such that health knowledge and behaviors targeted by the app’s messages will be measured at baseline. These questions will be developed around domains of knowledge such as healthy eating and avoiding smoking. Next, the same knowledge and behaviors will be measured after use of the 4YYB or control app, with the time frame specified to start when the user starts using the app. This will facilitate evaluating whether the 4YYB messages have a differential impact on knowledge and behaviors in different domains.

This app, once developed and tested, can prove useful for dissemination of healthy pregnancy knowledge and behaviors in other Middle Eastern countries where Arabic is the main spoken language. These countries are predominantly Islamic, so messages would already be culturally acceptable. The other countries on the Arabian Peninsula, such as Qatar, Yemen, United Arab Emirates, and Kuwait, would likely easily be able to adopt this app as these countries are more affluent and tend to have higher rates of smartphone use. However, a leaner text-only version could be used in Egypt, which is a low- to middle-income country where cell phone use (but not necessarily smartphone use) is prevalent.

### Conclusions

As KSA seeks to improve its pregnancy outcomes, it can leverage Saudi women’s natural adoption of smartphone apps as a communication channel for delivering pregnancy health information by way of an Arabic-language mobile app adapted from Text4Baby. Although messages will need to be adapted and the app redesigned and enhanced, the development of 4YYB offers the opportunity for developing a rigorous evidence base behind the efficacy of the mobile app. By introducing the use of a control message app and using questions targeting knowledge in the apps domains, studies of 4YYB should provide clear evidence of the differential impact of the app’s messages on healthy pregnancy knowledge and behaviors in Saudi Arabian women.

## Abbreviations

4YYB: For You and Your Baby
CDC: United States Centers for Disease Control and Prevention
KAUH: King Abdulaziz University Hospital
KSA: Kingdom of Saudi Arabia
NGHA: Ministry of National Guard Health Affairs (Saudi Arabia)
OB/GYN: obstetrics and gynecology
RCT: randomized controlled trial
SES: socioeconomic status
SMS: short message service
SPTB: spontaneous preterm birth
VLBW: very low birthweight
